# Factors associated with environmental barriers of people with disabilities in Mexico

**DOI:** 10.11606/S1518-8787.2019053000556

**Published:** 2019-03-27

**Authors:** Liliana Giraldo-Rodríguez, Dolores Mino-León, Juana Catalina Murillo-González, Marcela Agudelo-Botero

**Affiliations:** I Instituto Nacional de Geriatría, Subdirección de Investigación Epidemiológica Geriátrica. Departamento de Epidemiología Demográfica y Determinantes Sociales. Ciudad de México, México; II Instituto Mexicano del Seguro Social. Unidad de Investigación en Epidemiología Clínica. Hospital de Especialidades. Coordinación de Investigación en Salud. Ciudad de México, México; III Secretaría de Gobernación. Subsecretaría de Prevención y Participación Ciudadana. Ciudad de México, México; IV Universidad Nacional Autónoma de México. Facultad de Medicina. Centro de Investigación en Políticas, Población y Salud. Ciudad de México, México

**Keywords:** Disabled People, Self-Assessment, Activities of Daily Living, Environmental Design, Socioeconomic Factors, Cross-Sectional Studies

## Abstract

**OBJECTIVE:**

To examine the associations between sociodemographic, health and disability-related factors and the perception of environmental barriers outside the home environment by individuals with permanent disabilities in Mexico.

**METHODS:**

In this cross-sectional, population-based study, we used data from the 2010 National Survey of Perceptions of Disability in the Mexican Population of 2,041 participants older than 18 with permanent disability. The perceptions of barriers take into consideration the challenges of getting around and using transportation outside the home environment. The covariates consisted of sociodemographic, health-related and disability-related factors. Multivariate logistic regression was used.

**RESULTS:**

The perception of environmental barriers outside the home environment was associated with being a woman, living in an urban area, speaking an indigenous language, experiencing emotional symptoms, having walking/movement, visual or self-care disabilities, having severe/extreme disability, having disability caused by illness, using physical devices, and receiving assistance and care in the home environment.

**CONCLUSIONS:**

This information is valuable for the design of public policies and programs that promote the participation of individuals with permanent disabilities, a high-priority issue in low- and middle-income countries.

## INTRODUCTION

According to the International Classification of Functioning, Disability, and Health (ICF), disability is the result of a complex relationship between a person's state of health and his or her personal, contextual, and environmental characteristics, including the circumstances in which he or she lives^1^ ^,^ ^2^. Environmental factors make up the physical, social and attitudinal environment in which people live, including accessibility barriers and social policies, and can affect the performance of an individual's day-to-day activities in negative or positive ways^1^ ^,^ ^3^ ^–^ ^5^. These barriers include all the factors in a person's environment that, due to their absence or presence, limit functioning and create disability^1^. The environmental barriers outside the home environment that are most frequently encountered by individuals with disabilities are lack of architectural structures (e.g., ramps, elevators)^6^ ^–^ ^8^; inadequate or poor visual, tactile or acoustic signaling^9^; and lack of adapted transportation^10^ ^–^ ^12^, among others.

The context in which individuals with disabilities live may or may not facilitate their participation in different activities, such as studying, working, visiting a doctor, or practicing sports. In the literature, it has been suggested that the environmental barriers can be more significant than the disability itself^2^ ^,^ ^4^ ^,^ ^6^ ^,^ ^13^. These barriers can cause loss of autonomy and increase dependency^2^ ^,^ ^14^. Individuals with disabilities who experience environmental barriers are at higher risk of accidents, including falls and fractures^15^ ^–^ ^17^; are less physically active^18^ ^,^ ^19^; spend more time at home; are more likely to be overweight and obese^20^; and are more likely to experience chronic illnesses^15^ ^,^ ^21^ ^–^ ^23^.

The problems of participation in daily activities and disability-related health outcomes may be the result of a person's limited physical capacity, the presence of environmental barriers and the absence of facilitators, or a combination of all of them. The ICF recognizes the key role of environmental factors, recommending that they should be analyzed from the perspective of individuals with disabilities to design intervention strategies that promote their inclusion in society^1^.

In recent years, researches have become increasingly focused on identifying the environmental factors that promote or restrict the participation of individuals with disabilities in society and the effects on their health and well-being. Few studies have focused on identifying how personal, environmental, and health-related factors are associated with the perception of barriers. Most of these studies have been conducted in developed countries, whereas this type of research is still emerging in middle- and low-income countries^24^. Consequently, the purpose of this study was to identify sociodemographic, health, and disability-related factors associated with the perception of environmental barriers outside the home environment by individuals with permanent disabilities in Mexico.

## METHODS

### Study Design and Data Collection

The data were collected from a cross-sectional study of the 2010 National Survey of Perceptions of Disability in the Mexican Population (*Encuesta Nacional de Percepción de la Discapacidad en Población Mexicana* – ENPDis, in Spanish)^25^, conducted by Mexico's Health Ministry and National Public Health Institute (NPHI). One of the objectives of the survey was to describe the factors that promote or restrict the access of individuals with disabilities to public spaces and agencies. The data analysis was performed in 2015.

The sample was probabilistic, multistage, stratified, and clustered, with nationwide, urban, and rural representation. A total of 5,423 households were visited (2,216 rural and 3,207 urban). Four questionnaires were applied (namely, to households, individuals with disabilities, and individuals without disabilities, as well as regarding the characteristics of the city or town) by a staff previously trained in a standardized manner. Direct interviews were conducted with individuals older than 10. In the ENPDis, the questions asked to individuals with permanent disability were based on the recommendations of the Washington Group^25^; the questions used were the following: “In your everyday life, do you have trouble: walking, getting around, moving up or down?; seeing (even when using glasses)?; speaking, communicating or conversing; hearing (even when using a hearing aid)?; dressing, bathing or eating?; or paying attention or learning simple things?; as well as, do you have any mental disabilities?”

### Participants

The participants consisted of all individuals identified by the household informants (via the household questionnaire) as well as those with permanent disabilities (i.e., disabilities lasting longer than six months). In this study, an individual with a permanent disability was defined as someone who described experiencing impairment in at least one of the following functional domains: walking, getting around, moving up or down, seeing (even when using glasses), speaking, communicating or conversing, hearing (even when using a hearing aid), dressing, bathing or eating without assistance, or paying attention or learning simple things, or someone who had had a mental disability for at least six months^25^. The ENPDis contains information from 3,443 individuals with permanent disabilities, but we included only individuals who performed some type of activity outside their homes (n = 2,041).

### Measures

Dependent variable: Environmental barriers outside the home environment was a variable based on the difficulty reported by individuals when moving or commuting to a health or rehabilitation center, school, or workplace, or when participating in any family, community, sports, or cultural activities. The presence of barriers outside the home environment was measured using a dichotomous variable (0 = no barrier; 1 = with a barrier).

Covariates: sociodemographic factors included the following: sex (female, male); age (18 to 59, ≥ 60 years); marital status (single [no partner], partnered); place of residence (rural, urban); education (< 10 years, ≥ 10 years); and spoken use of an indigenous language (yes, no).

Health-related factors included the following: self-perception of one's state of health (very good or good, regular, poor or very poor); and emotional symptoms (question: “During the last 30 days, to what degree have you felt sad, downcast, or depressed?” answers: present, absent).

Disability-related factors included the following: self-report of one's primary disability (walking or mobility, visual, hearing, speaking or communication, attention or learning, or self-care; answers were analyzed as dichotomous variables); number of difficulties (answers were classified as the following: one difficulty, two or more difficulties); degree of severity of the primary disability (difficulty that the person attributed to their primary disability when carrying out daily activities, with answers categorized as follows: mild, moderate, severe or extreme); cause of the primary disability (the cause of the disability as described by the individual, with answers categorized as follows: illness, accident, old age, birth, and other); physical devices (questions: “As a result of your difficulties walking, do you use: crutches, a wheel chair, a walker, a cane, or any other device?”; “Because of your difficulties seeing, do you use a white cane, braille, or another tool?”; answers: uses, does not use); prosthesis or orthosis (questions: “Do you use any type of prosthesis – that is, has something been attached to your body to replace a body part?” and “Do you use a device or tool to help you better use any part of your body?”; answers: uses, does not use); assistance and care in the home environment (question: “Do you receive assistance or personal care in your home because of your difficulty?”; answers: receives, does not receive).

### Data Analysis

Frequencies and percentages were calculated according to each type of variable. A bivariate analysis was conducted using a chi-squared test, with statistical significance p < 0.05, to determine whether a statistical association exists between the variables and the perception of environmental barriers outside the home environment. A multivariate logistic regression model was used to analyze the covariates associated with the perception of environmental barriers outside the home environment, and odds ratios (OR) and 95% confidence intervals were obtained. Chi-squared, Wald, and p-value tests were used to measure the significance of the individual model parameters, and the Hosmer-Lemeshow chi-squared goodness-of-fit test was used to estimate the overall fit of each model. The model was adjusted for the covariates that were found to be significant during the bivariate analysis. The data were analyzed using the complex sampling function of Stata 11.0 (Stata Corp, College Station, Texas, USA), and p ≤ 0.05 was used to define statistical significance.

### Ethical Considerations

The ENPDis was approved by the NPHI Ethics Committee. An informed consent form was used. The database is publicly available and does not contain the individuals' identification.

## RESULTS

The mean age of the individuals with permanent disabilities who served as participants in this study (n = 2,041) corresponded to 58.1±17.1 years old, with 47% being between 18 and 59 years of age; 52.6% were women, and 47.4% did not have a partner at the time of the interview. Most of the individuals with permanent disabilities lived in urban areas (78.3%); 82.7% had < 10 years of education, and 8.9% spoke an indigenous language. Approximately 36% of the individuals with disabilities perceived their health as very good or good, and 49.5% perceived it as average. Additionally, 60.7% reported having emotional symptoms. The most commonly self-reported primary disability involved walking or movement (62.4%), followed by visual (23.0%), hearing (5.7%), speaking and communicating (4.2%), attention and learning (3.2%), and self-care (1.5%) disabilities. A single disability was reported by 69.1% of the participants, whereas 30.9% had ≥ 2 disabilities. About the severity of the primary disability, 55.3% described it as severe or extreme. The most commonly self-reported cause of primary disability was illness (33.1%), followed by accident (24.8%), old age (15.5%), and birth (13.4%). In total, 39.0% used physical devices, and 14.5% used a prosthesis or orthosis. Only 27.3% described receiving assistance or care in the home environment (Table 1).

**Table 1:** Characteristics of individuals with permanent disabilities.

Variable		Frequency	Percentage
Sex			
	Female	1,074	52.6
	Male	967	47.4
Age (years old)			
	18 to 59	959	47.0
	≥ 60	1,082	53.0
Marital status			
	Single (no partner)	968	47.4
	Partnered	1,073	52.6
Place of residence			
	Rural	443	21.7
	Urban	1,5981	78.3
Education level (years)			
	< 10	1,687	82.7
	≥ 10	354	17.3
Speaks an indigenous language			
	Yes	181	8.9
	No	1,860	91.1
Self-perceived state of health			
	Very good or good	729	35.7
	Regular	1,010	49.5
	Poor or very poor	302	14.8
Emotional symptoms			
	Present	1,239	60.7
	Absent	802	39.3
Self-reported primary disability			
	Walking or mobility	1,274	62.4
	Visual	469	23.0
	Hearing	117	5.7
	Speaking or communication	85	4.2
	Attention or learning	65	3.2
	Self-care	31	1.5
Number of difficulties			
	1	1,410	69.1
	≥ 2	631	30.9
Degree of severity of the primary disability			
	Mild	236	11.6
	Moderate	677	33.2
	Severe or extreme	1,128	55.3
Cause of primary disability			
	Illness	676	33.1
	Accident	507	24.8
	Old age	316	15.5
	Birth	273	13.4
	Other	183	9.0
	Unknown	86	4.2
Physical devices			
	Uses	795	39.0
	Does not use	1,246	61.1
Prosthesis or orthosis			
	Uses	295	14.5
	Does not use	1,746	85.6
Assistance and care in the home environment			
	Receives	557	27.3
	Does not receive	1,484	72.7

A total of 60.9% of the individuals interviewed perceived environmental barriers outside the home environment. The variables that showed greater strengths of association and statistical significance were the following: self-reported primary disability, degree of severity of the primary disability, physical devices, cause of primary disability and home care (Table 2).

**Table 2:** Comparison between groups with and without perception of environmental barriers outside the home environment. (n = 2,041)

Variable		Perception of environmental barriers outside the home environment				
		Yes (n = 1,243)	No(n = 798)	Chi-square	p	Cramer's V
		n (%)	n (%)			
Sex						
	Female	675 (54.3)	399 (50.0)	3.61	0.057	0.042
	Male	568 (45.7)	399 (50.0)			
Age (years old)						
	18 to 59	524 (42.2)	435 (54.5)	29.78	0.000	0.121
	≥ 60	719 (57.8)	363 (45.5)			
Marital status						
	Single (no partner)	603 (48.5)	365 (45.7)	1.50	0.221	0.027
	Partnered	640 (51.5)	433 (54.3)			
Place of residence						
	Rural	292 (23.5)	151 (18.9)	5.97	0.015	0.054
	Urban	951 (76.5)	647 (81.1)			
Education (years)						
	< 10	1,050 (84.5)	637 (79.8)	7.32	0.007	0.060
	≥ 10	193 (15.5)	161 (20.2)			
Speaks an indigenous language						
	Yes	140 (11.3)	41 (5.1)	22.56	0.000	0.105
	No	1,103 (88.7)	757 (94.9)			
Self-perceived state of health						
	Very good or good	397 (31.9)	332 (41.6)	58.44	0.000	0.169
	Regular	605 (48.7)	405 (50.8)			
	Poor or very poor	241 (19.4)	61 (7.6)			
Emotional symptoms						
	Present	849 (68.3)	390 (48.9)	76.92	0.000	0.194
	Absent	394 (31.7)	408 (51.1)			
Self-reported primary disability						
	Walking or mobility	883 (71.0)	391 (49.0)	248.69	0.000	0.349
	Visual	276 (22.2)	193 (24.2)			
	Hearing	7 (0.6)	110 (13.8)			
	Speaking or communication	26 (2.1)	59 (7.4)			
	Attention or learning	23 (1.9)	42 (5.3)			
	Self-care	28 (2.3)	3 (0.4)			
Number of difficulties						
	1	826 (66.5)	584 (73.2)	10.31	0.001	0.071
	≥ 2	417 (33.5)	214 (26.8)			
Degree of severity of the primary disability						
	Mild	67 (5.4)	169 (21.2)	195.47	0.000	0.310
	Moderate	353 (28.4)	324 (40.6)			
	Severe or extreme	823 (66.2)	305 (38.2)			
Cause of primary disability						
	Illness	488 (39.3)	188 (23.6)	126.00	0.000	0.249
	Accident	299 (24.1)	208 (26.1)			
	Old age	211 (17.0)	105 (13.2)			
	Birth	109 (8.8)	164 (20.6)			
	Other	110 (8.8)	73 (9.1)			
	Unknown	26 (2.1)	60 (7.5)			
Physical devices						
	Uses	634 (51.0)	161 (20.2)	194.26	0.000	0.309
	Does not use	609 (49.0)	637 (79.8)			
Prosthesis or orthosis						
	Uses	186 (15.0)	109 (13.7)	0.67	0.413	0.018
	Does not use	1,057 (85.0)	689 (86.3)			
Assistance and care in the home environment						
	Receives	432 (34.8)	125 (15.7)	89.26	0.000	0.209
	Does not receive	811 (65.2)	673 (84.3)			

Table 3 shows the results of the logistic regression model. The sociodemographic and health variables that were significantly associated with the perception of environmental barriers outside the home environment for individuals with permanent disabilities were as follows: being female; living in an urban area; speaking an indigenous language; presently experiencing emotional symptoms; having a primary disability related to walking or mobility, visual, speaking or communication or self-care; having a moderate, severe, or extreme degree of severity of the primary disability; having a disability caused by illness or old age; using physical devices; and receiving assistance and care in the home environment. The perception of environmental barriers outside the home environment was lower in cases of hearing disabilities (OR = 0.19, p < 0.05).

**Table 3:** Factors associated with the perception of environmental barriers outside the home environment by individuals with permanent disabilities in Mexico.

Variable		Odds ratio	95%CI	p
Sex (reference: male)				
	Female	1.45	1.2–1.8	< 0.001
Age (reference: 18 to 59 years old)				
	≥ 60	1.11	0.9–1.4	0.43
Place of residence (reference: rural)				
	Urban	1.68	1.3–2.2	< 0.001
Education (reference: < 10 years)				
	≥ 10 years	1.17	0.9–1.6	0.34
Speaks an indigenous language (reference: no)				
	Yes	1.82	1.2–2.8	< 0.05
Self-perceived state of health (reference: very good or good)				
	Average	0.78	0.6–1.0	< 0.05
	Poor or very poor	1.43	0.9–2.2	0.09
Emotional symptoms (reference: absent)				
	Present	1.61	1.3–2.0	< 0.001
Self-reported primary disability (reference: attention or learning)				
	Walking or mobility	4.33	2.3–8.1	< 0.001
	Visual	2.82	1.5–5.3	< 0.001
	Speaking or communication	2.23	1.0–4.9	< 0.05
	Hearing	0.19	0.1–0.5	< 0.001
	Self-care	14.39	3.5–58.9	< 0.001
Number of difficulties (reference: 1)				
	≥ 2	1.01	0.8–1.3	0.94
Degree of severity of the primary disability (reference: mild)				
	Moderate	2.39	1.6–3.5	< 0.001
	Severe or extreme	4.58	3.2–6.6	< 0.001
Cause of primary disability (reference: birth)				
	Illness	2.09	1.4–3.1	< 0.001
	Accident	1.08	0.7–1.6	0.69
	Old age	1.70	1.1–2.6	< 0.05
	Other	1.04	0.7–1.6	0.86
	Unknown	0.43	0.2–0.8	< 0.05
Physical devices (reference: does not use)				
	Uses	1.77	1.4–2.3	< 0.001
Assistance and care in the home environment (reference: does not receive)				
	Receives	1.86	1.4–2.4	< 0.001

## DISCUSSION

This is the first study at national level in Mexico to describe sociodemographic, health-related and disability-related factors and examine their association with the perception of environmental barriers outside the home environment by individuals with permanent disabilities. One of the study's findings revealed that some of the disability-related factors (type, severity, cause, need for assistance, and use of devices) were more strongly associated with the perception of environmental barriers outside the home environment. This last finding corroborates those of previous studies, in which physical limitations (self-care and mobility) and the degree of disability were associated with a greater perception of environmental barriers^24^ ^,^ ^26^ ^,^ ^27^.

Detecting an association between the degree of severity and the primary cause of the disability (illness or old age) with a higher perception of environmental barriers outside the home environment leads us to believe that individuals who lose physical functionality need to acquire new abilities to adapt to their new condition, and create compatible surrounding environments. In this sense, new interventions should consider the individual's physical abilities in various areas of his or her environment, so that rehabilitation can serve to increase this individual's functional capacity by correcting or improving the limitations detected. Health services should be particularly sensitive to the needs of older people who have lost functionality. Providing appropriate services to older adults with disabilities is a priority for WHO, as mentioned in the study on global aging and adult health (SAGE)^28^.

The results of this study also showed that individuals who use physical devices (crutches, wheel chair, walker, cane, or any other device) have a greater perception of environmental barriers outside the home environment. This finding is similar to those reported in a previous study, which showed that the use of physical devices by individuals with limitations in their lower extremities is associated with a greater perception of barriers^29^. According to the above, we can consider that the physical context and environment in which individuals with disabilities live can adversely affect their activities outside the home environment, but this outcome is also observed in individuals who use physical devices designed to improve their mobility, which is a fact worth noting and that needs to be further studied.

Another finding was the significant association of emotional symptoms with the perception of environmental barriers outside the home environment, which has already been reported in the literature, showing that emotional symptoms increase the risk of social isolation, loneliness and depression^30^.

Our results also show that individuals with permanent disabilities living in urban areas have a greater perception of barriers than those living in rural areas. This finding could be explained by the complexity of Mexican urban contexts, which are characterized by high population density, excessive concentration of services, and poor urban planning^11^. All these factors would, at least in our country, make cities unsuitable for individuals with physical or mental limitations, because their needs are not being met. However, this finding should be explored in detail, because this study only included individuals with disabilities venturing out of their homes, while not exploring reasons why other individuals with permanent disabilities decided not to do so^31^.

Another finding that should be explored in detail is the association between speaking an indigenous language and the perception of environmental barriers, which is relevant in Mexico because the prevalence of disability in the indigenous population older than three years of age is 7.1%, which is higher than the national prevalence (6%). These data suggest that the indigenous population is considered to be more vulnerable, which may be associated with increased negative perception of the environment and being exposed to more physical obstacles to movement outside the home environment^32^.

This study has some limitations, some of which are related to the type of information collected in the ENPDis. For example, the survey only contained information about environmental barriers existing outside the home environment that were perceived by individuals with permanent disabilities. The existence of other types of barriers, including social and attitude-related challenges, have been described elsewhere in the literature^3^ ^,^ ^31^ ^,^ ^33^ ^–^ ^36^, and these barriers negatively affect this group's participation in various day-to-day activities. Another limitation is that the survey only addressed barriers that exist outside the home environment, and information is not available regarding household barriers to the performance of basic and essential activities. In this sense, what is needed is a study identifying the physical, social and attitudinal factors in the environments in which people lead their daily lives, to see if these factors represent barriers that affect their physical capability to perform various basic everyday activities. It is also necessary to incorporate a greater number of personal factors associated with lifestyle into the study such as habits and life experience, that can also affect an individual's perception of environmental barriers. Another limitation of this study is that it is a cross-sectional study; therefore, no causal relationship between the factors reported and the perception of barriers can be assumed.

## CONCLUSION

The perception of environmental barriers outside the home environment is primarily associated with disability-related factors, as well as some sociodemographic and health-related factors. We believe that this information is very valuable because, for the first time in Mexico, the obstacles in the physical environment for people with functional limitations are made clear. These obstacles create conditions that magnify their disability. In Mexico, the findings of this study can be considered in the evaluation and improvement of public policies currently in effect, such as the General Law for the Inclusion of People with Disabilities, as well as the process of adapting the National Development Program to this law. The development of institutional actions to ensure the full rights of people with disabilities can also take these findings into account.
